# Recent Advances in Research on Widow Spider Venoms and Toxins

**DOI:** 10.3390/toxins7124862

**Published:** 2015-11-27

**Authors:** Shuai Yan, Xianchun Wang

**Affiliations:** Key Laboratory of Protein Chemistry and Developmental Biology of Ministry of Education, College of Life Sciences, Hunan Normal University, Changsha 410081, China; acemalcolm@163.com

**Keywords:** widow spider, venom, toxin, latrotoxin, latroeggtoxin, advance

## Abstract

Widow spiders have received much attention due to the frequently reported human and animal injures caused by them. Elucidation of the molecular composition and action mechanism of the venoms and toxins has vast implications in the treatment of latrodectism and in the neurobiology and pharmaceutical research. In recent years, the studies of the widow spider venoms and the venom toxins, particularly the α-latrotoxin, have achieved many new advances; however, the mechanism of action of the venom toxins has not been completely clear. The widow spider is different from many other venomous animals in that it has toxic components not only in the venom glands but also in other parts of the adult spider body, newborn spiderlings, and even the eggs. More recently, the molecular basis for the toxicity outside the venom glands has been systematically investigated, with four proteinaceous toxic components being purified and preliminarily characterized, which has expanded our understanding of the widow spider toxins. This review presents a glance at the recent advances in the study on the venoms and toxins from the *Latrodectus* species.

## 1. Introduction

*Latrodectus* spp. belong to the family Theridiidae (Arthropoda, Arachnida, Araneae) [1,2]. The genus contains more than 30 species that are distributed worldwide, including China, Central Asia, Southern Europe, North and South America, India, and Australia [3,4,5]. The *Latrodectus* spp. are highly venomous [6] and responsible for a number of spider bites and envenomations around the world [7]. *Latrodectus* spp. venoms have been studied extensively, initially focusing on *L.* *tredecimguttatus* and *L.* *mactans* [5,8], but more recently on other *Latrodectus* spp. venoms [3]. *Latrodectus* spp. are called widow spiders because some females eat the male after mating [9]. Widow spiders are most often black—hence the common name “black widow”—and usually have a red hourglass on the ventral side of their abdomen. The large venom glands and long fangs of the female black widow spiders, commonly used for predation and defense, are adequate to constitute a danger to human beings and, therefore, many cases of envenomation have been presented [10]. The distant part of each chelicerae is a mobile hollow fang that penetrates the skin during a bite, injecting venom into the prey [11]. Envenomation by the spider causes neurotoxic symptoms such as sharp pain around the site of bite, whereafter diaphoresis, hypertension, patchy paralysis, *etc.*, may occur [3]. Although death is rare, remarkable and distressing discomfort is representative [12], and in many situations a systematic manifestation called latrodectism was developed [13] which is thought to be associated with an exhaustive release of the neurotransmitters, especially norepinephrine and acetylcholine, due to spider envenomation [14,15]. For many decades, antivenom has been considered an effective treatment of latrodectism [16]. However, in a recent report, Isbister *et al.* [17] claimed that antivenom was no more effective than placebo in treating latrodectism in Australia, which is at odds with both the past literature and extensive independent clinical experience and needs to be further confirmed [16].

Interestingly, different from many other venomous animals including snakes and some other spider species that have toxins only in their venom glands, black widow spiders have toxins not only in their venom glands, but also throughout their body, including in the legs and abdomen, and even in the eggs and newborn spiderlings [18,19,20,21], which is speculated to be helpful for increasing the probability of individual survival and species continuation. The black widow spider materials, including the venom, eggs, and spider body tissues, can be deemed as a valuable library of biologically active molecules. Inquiries into the venoms and toxins have major significance not only in treatment of the latrodectism, but also in pharmaceutical research and tool reagent development which is supposed to be helpful to elucidate pathological and physiological processes. This review presents a glance at the main advances in recent years in the study of widow spider venoms and toxins.

## 2. Physiological and Biochemical Analysis of Venoms

### 2.1. Physiological Analysis

The venom secreted by widow spider venom glands is a complex mixture of components with diverse biological functions. Many of them are biologically active proteins and peptides, which play a number of adaptive roles: paralyzing, immobilizing, killing, liquefying prey, and restricting competitors [5]. From early times there have been sporadic reports on the toxicity of the venom of black widow spiders [5,22]. In recent years, the systematic analyses of black widow venoms have deepened our understanding of the spider toxicity. For example, before long, the effect of a Chilean black widow spider (*L. mactans*) venom on bovine spermatozoa was investigated. The results indicated that the venom increased the Ca^2+^ influx with an EC_50_ of 6.1 μg/mL and triggered the acrosome reaction in 43.26% of the cells. The application of potassium (10 mM K^+^) or venom (10 μg/mL) did not affect the morphology or DNA stability of the sperm. The effects induced by high K^+^ and venom suggest that direct blocking of K^+^ currents alters the passive properties of the plasma membrane, leading to the entry of Ca^2+^. These results show the importance of functional changes induced by depolarizing the spermatozoa and by the venom. This venom possesses one or more molecules that may be used as pharmacological tools for studies on spermatozoa and have potential applications in reproductive biotechnology [23,24]. When the brown widow spider (*L. geometricus*) venom was investigated, it was found that the venom damaged the adrenal gland, producing severe alterations on cortex cells and resulting in death by acute adrenal insufficiency. The venom was confirmed to contain fibrinogenolytic and other proteolytic activities, showing specific actions on extracellular matrix proteins such as fibronectin, laminin, collagen type IV, and fibrinogen that might play certain roles in the spider toxicity [25]. Wang *et al.* [26] employed multiple physiological and biochemical strategies to systematically analyze the electrical stimulation-collected black widow spider (*L. tredecimguttatus*) venom that was not contaminated with histiocytic proteins and other components and, therefore, was more representative of the pure venom. The venom was demonstrated by gel electrophoresis and mass spectrometry to consist primarily of proteins with molecular masses above 10 kDa, most of which are high-molecular-mass acidic proteins, with fewer proteins and peptides below 10 kDa. The most abundant proteins are distributed around 100 kDa. The venom was demonstrated to be rich in neurotoxins because injection of the venom in mice and *P. americana* led to obvious poisoning symptoms, with LD_50_ values of 0.16 mg/kg and 1.87 μg/g, respectively. In addition, the venom could efficiently block the neuromuscular transmission in isolated mouse phrenic nerve-hemidiaphragm and rat vas deferens preparations, and the low-molecular-mass fraction (<10 kDa) of the venom had no obvious effect on the transmission, suggesting that the mammalian toxicity of the venom is primarily based on its larger proteins. Enzymatic analysis indicated that the venom contains multiple kinds of hydrolases, including proteinases, hyaluronidase, alkaline, and acid phosphatases. All of the data demonstrate that the venoms are rich in ion channel modulators and metabolic enzymes, particularly the proteolytic enzymes that can enhance the action of the toxins by breaking down the intercellular reinforcements and basement membrane molecules.

### 2.2. Whole Venom Proteomics

The complexity of black widow spider venom has long been appreciated by researchers in the fields of toxicology and medicine. However, it is the recent advances in protein separation and biological mass spectrometry that allow virtually all venom components to be effectively separated and identified. In 2008, Duan *et al.* [27] employed a combinative strategy to make a proteomic analysis of the venom collected from living adult spiders (*L. tredecimguttatus*) by electrical stimulation. A total of 122 non-redundant venom proteins were unambiguously identified, 75 of which had distinct function annotation. Besides the previously reported widow spider venom proteins including latrotoxins, a variety of hydrolases and other proteins with special activity were found in the venom, such as proteinase, phospholipase, phosphatase, nuclease, fucolectin, venom allergen antigen 5-like protein, and trypsin inhibitor. These results help to understand the complexity and action mechanism of black widow spider venom. Up until now, the mechanism of action of the black widow spider venom has not been completely clear. It was speculated that the intoxication after a bite by a black widow spider must be related to, besides latrotoxins, other venom components such as hydrolases. Hydrolases have also been identified in the venoms of several snakes and other spider species, and some hydrolases, particularly the proteases such as metalloproteases and serine proteases, were demonstrated to have some participation in the noxious effects of the venoms [28,29,30]. Obviously, the hydrolases and other proteins with special activity in the black widow spider venom should have important potentials in enhancing venom toxicity and extending the venom action scope and model. In addition, there are 47 proteins being matched to predicted protein, hypothetical protein, and protein of unknown function, respectively. These proteins are also potential active components of the venom, which need to be further confirmed.

In an attempt to uncover the dramatic expansion of the black widow toxin arsenal, Haney *et al.* [31] used a proteomic strategy to determine the venom proteins from the Western black widow spider (*L. hesperus*). Sixty-one proteins were identified with the mass spectrometry technique from an *L. hesperus* protein database that matched peptides collected from *L. hesperus* venom, including 21 latrotoxins, one ICK (inhibitor cystine knot) toxin, and six CRISP (cysteine-rich secretory protein) family toxin proteins. Several types of enzymes were identified in the venom, including hyaluroidases, chitinase, serine proteases, and metalloproteases. These results demonstrate that *L. hesperus* and *L. tredecimguttatus* have similarities in the molecular basis of venom toxicity.

## 3. Venom Gland Transcriptome

To more comprehensively analyze the proteinaceous components in the venom, venom gland transpcriptomes have been paid much attention recently. In 2013, a venom gland transcriptome of the spider *L. tredecimguttatus* was constructed by using a combination of next-generation sequencing and conventional DNA sequencing, which resulted in the identification of 9666 and 480 high-confidence protein coding transcripts among 34334 *de novo* sequences and 1024 cDNA sequences, respectively. Functional analyses indicated that mRNAs involved in RNA transcription and spliceosome, protein translation, processing, and transport are highly enriched in the venom glands. Among the high-confidence proteins, 146 were demonstrated to be toxin-like proteins forming 12 families: α-LTX-Lt1a family 1, α-LTX-Lt1a family 2, α-LIX-Lt1a family, δ-LIX-Lt1a family, Ank family, Theriditoxin family, SCP family, Ctenitoxin family, Trypsin family, Lycotoxin family, Orphan family, and Scorpion toxin like family (Table 1). All six known toxins from *L. tredecimguttatus* and homologues to 16 known toxins from other species were included in the identified transcriptome dataset. The 146 toxin-like proteins were categorized into five classes according to their bioactivities: neurotoxins, assistant toxins, proteases, protease inhibitors, and unknown function toxins. The authors discussed how these toxins work cooperatively in latrodectism: neurotoxins act as the main toxic components and specifically target the nervous system; assistant toxins may enhance the toxicity of neurotoxins; protease inhibitors protect neurotoxins and assistant toxins from proteolytic degradation; and the proteases may aid in the maturation of toxin precursors and help to digest the prey [32]. In addition, the comparison of the results of the sequence analysis with those of the above-mentioned proteomic identification demonstrates that many toxins identified by sequence analysis, particularly the latrotoxins, enzymes, and enzyme inhibitors, had been identified at the protein level, which provides comprehensive information on the molecular basis of the venom toxicity.

**Table 1:** Toxins identified by sequence analysis from *L. tredecimguttatus* venom gland.

Family Name	Number of Member	Function/Activity
α-LTX-Lt1a family 1	12	main neurotoxins against vertebrates
α-LTX-Lt1a family 2	7	main neurotoxins against vertebrates
α-LIX-Lt1a family	2	main neurotoxins against insects
δ-LIX-Lt1a family	6	main neurotoxins against insects
Ank family	4	neurotoxins
Theriditoxin family	62	assistant toxins
SCP family	3	ion channel inhibitors
Ctenitoxin family	9	protease inhibitors
Trypsin family	16	toxin maturation; hydrolysis of prey tissues
Lycotoxin family	8	neurotoxins
Orphan family	13	inhibitors of proteases or ion channels
Scorpion toxin like family	4	largely unknown

More recently, Haney *et al.* [31] combined next-generation RNA sequencing and bioinformatic analysis to determine venom gland-specific transcripts from the Western black widow spider (*L. hesperus*) and investigate their evolution. They identified 695 venom gland-specific transcripts (VSTs). Up to 38% of the VSTs had BLAST (basic local alignment search tool) hits, including latrotoxins, ICK toxins, CRISPs, hyaluronidases, chitinase, and proteases, and 59% of the VSTs had predicted protein domains. Among the VSTs there were 45 with significant BLAST similarity to known venom toxins and 17 enzymes that may act to facilitate toxin action. The analysis of *L. hesperus* toxin diversity and evolution indicated that the majority of the diversity among the VSTs with BLAST homology to known toxins was contributed by latrotoxins. A total of 39 VST sequences were identified as latrotoxins. Low-molecular-mass ICK toxins were previously not considered to be a part of the *Latrodectus* spp. venom [33]. However, the transcriptomic analysis revealed that the sequences encoding these small peptides were also present among the transcriptomes of *L. tredecimguttatus* [32] and *L. hesprus* [31]. Among the venom gland-specific transcript set, overall expression is dominated by putative neurotoxins and their associated molecules, although they make up only a minority of the distinct transcripts. Generally speaking, there are many similarities between the transcriptomes of *L. hesperus* and *L. tredecimguttatus,* particularly in the types of toxins, hydrolases, and inhibitors. However, variation was evident when comparing *L. hesperus* protein sequences with the functionally characterized orthologs from *L. tredecimguttatus*. For example, the *L. hesperus* sequence venom_comp106397_c0_seq groups closely with *L. tredecimguttatus* δ-latroinsectotoxin, but has 14 ankyrin repeats, as opposed to 13 in the sequence from *L. tredecimguttatus* [34]. The inferred functions of the identified transcript sequences indicate that the venom of black widow spiders is extremely diverse at the molecular level, and is the product of a complex evolution history.

## 4. Toxins Purified from Venom

### 4.1. Main Venom Toxins

Up until now, at least seven different latrotoxins (LTX) have been isolated from the venom of the *L. tredecimguttatus* spider by means of techniques such as ion exchange chromatography and hydrophobic chromatography [35,36,37] (Table 2). All the studied latrotoxins are large acidic proteins (pI ~5.0–6.0) with molecular masses ranging from 110 to 140 kDa. Most of them are targeted against insects and are called latroinsectotoxins (LITs) (α, β, γ, δ, ε-LIT) [27]. ε-LIT is also highly toxic to *C. elegans* [38]. α- LTX is the only known venom component that aims specifically at vertebrates [35]. α-Latrocrustatoxin (α-LCT) is active only in crustaceans [37]. To date, the primary structures of four genes respectively encoding four latrotoxins, α-LTX, α-LIT, α-LCT, and δ-LIT, were determined using cDNA or intron-less genomic cDNA [34,39,40,41]. Using electron cryo-microscopy, the three-dimensional (3D) structure of the mature α-LTX monomer was found to contain three regions: the wing (majority of domain II), the body (one quarter of domain II and first 15–16 ankyrin repeats), and the head (*ca.* 4.5 *C*-terminal ankyrin repeats) [42]. All the latrotoxins cause massive release of neurotransmitters from the nerve terminals of the respective animals after binding to specific neuronal receptors [5]. In addition, Volkova *et al.* [43] isolated two low-molecular-mass proteins, LMWP and LMWP2, from black widow spider venom, and the two proteins were inactive to mammals and insects. These low-molecular-mass proteins are called latrodectins and appear to augment the neurotoxicity of latrotoxins, probably by increasing their affinity for the membrane target and reducing vertebrate phyla-specificity such that α-LTX becomes active in insects [8,44,45]. Using phylogenetic analyses and evidence from gene structure, McCowan and Garb [46] showed that latrodectin peptides from black widow spider venom are derived from the ecdysozoan superfamily of neuropeptides containing crustacean hyperglycemic hormones and ion transport peptides. In addition, Akhunov *et al.* [47,48] studied Kininase and two bradykinin-potentiating peptides of the *L. tredecimguttatus* venom. They characterized the Kininase as a thiol endopeptidase, which cleaves internal peptide bonds at the proline carboxyl end. The two bradykinin-potentiating peptides prolong depressor effects of bradykinin, stimulate histamine release from cells, and decrease blood pressure in rats. The data available indicate that *Latrodectus* spp. venoms contain a cocktail of toxins and other biologically active substances; however, many of them wait to be isolated and characterized.

**Table 2:** Information on the main toxins purified from the venom.

Component	MW(kDa)	Target/Activity	References
α-LTX	130	Vertebrates	[35]
α-LCT	120	Crustaceans	[37]
α-LIT	120	Insects	[36]
β-LIT	140	Insects	[36]
γ-LIT	120	Insects	[36]
δ-LIT	110	Insects	[36]
ε-LIT	110	Insects, *C. elegans*	[36,38]
LMWP	8	Increase toxicity of LTXs	[43]
LMWP2	9.5	Increase toxicity of LTXs	[43]

### 4.2. Diversity of α-LTX

The most studied latrotoxin is α-latrotoxin (α-LTX), which has a molecular mass of about 130 kDa and exerts a lethal effect in vertebrates by inducing a massive neurotransmitter release, acting both in the presence and in the absence of Ca^2+^ [49,50]. The clinical symptoms of latrodectism are mainly due to the presence of α-LTX homologs in the spider venom [51]. Western blotting with polyclonal anti-α-LTX antibody revealed that this 130 kDa band presents in the venoms of five tested *Latrodectus* species (*L. tredecimguttatus*, *L. hasselti*, *L. mactans*, *L. lugubris*, and *L. hesperus*) and these proteins have antigenic similarity. However, Western blotting with the monoclonal antibody derived from *L. tredecimguttatus* α-LTX found no binding with any proteins in venoms of the other four species, suggesting that there are important structural differences between α-LTXs in the venoms of different *Latrodectus* species [51]. It is generally accepted that α-LTX is present in the venoms of all *Latrodectus* species [51,52]. However, it was reported that the venom from the Chilean black widow spider (*L. mactans*) did not contain α-LTX [53]. This may be incorrect because more recently Garb *et al.* [4] demonstrated that α-LTX is also present in the venom of the spider by analyzing the molecular evolution of α-LTX. By combining 33 kb of *L. hesperus* genomic DNA with RNA-Seq, Bhere *et al.* [54] characterized the α-LTX gene and discovered a paralog 4.5 kb downstream. A 4 kb intron interrupts the α-LTX coding sequence, while a 10 kb intron in the 3′UTR (untranslated region) of the paralog may cause non-sense-mediated decay, which is contrary to previous findings in *L. tredecimguttatus* that no intron is found in the coding sequence of α-LTX [55]. Phylogenetic analysis confirms these divergent latrotoxins diversified through recent tandem gene duplications. Thus, latrotoxin genes have more complex structures, regulatory controls, and sequence diversity than previously proposed.

### 4.3. Action Mechanism and Application of α-LTX

It was found that α-LTX creates Ca^2+^-permeable channels in lipid bilayers, which involves toxin assembly into homotetrameric complexes that harbor a central channel [56]. The Ca^2+^ influx through the channels induced by α-LTX in the presynaptic membrane accounts for a large part of its effects. Three proteins in the plasma membrane, neurexin (NRX), latrophilin (LPH or CIRL), and protein tyrosine phosphatase σ (PTPσ), have been found to function as the receptors for α-LTX. After binding a receptor, α-LTX can exert its effects through two major mechanisms: (i) Ca^2+^-dependent action involving α-LTX insertion into the plasma membrane and pore formation; and (ii) Ca^2+^-independent action based on receptor-mediated signaling. The advances in the study on the structure and functions of latrotoxins, particularly α-LTX, greatly improved our understanding of the mechanisms regulating neurotransmitter release. For earlier reviews see Ushkaryov *et al.* [5,57], Rohou *et al.* [58], and Silva *et al.* [59].

Although much effort has been focused on the research of α-LTX, the mechanism of action of α-LTX has not been completely clear and remains the research hotspot in the related field. In order to further probe and distinguish the two action mechanisms of α-LTX, Deak *et al.* [60] employed a combination of gene knockout, electrophysiology, and the other related techniques to investigate the effects of α-LTX on the synaptic exocytosis of hippocampal neurons and showed that the Ca^2+^-independent release mechanism of α-LTX requires the synaptic SNARE (soluble *N*-ethylmaleimide-sensitive protein receptor) proteins synaptobrevin/VAMP (synaptic vesicle-associated membrane protein) and SNAP-25 (synaptosomal-associated protein-25), and, at least partly, the synaptic active zone protein Munc13-1. In contrast, the Ca^2+^-dependent mechanism of α-LTX-induced release utilizes a novel pathway of membrane fusion that does not require the classical synaptic fusion machinery, and thus differs from the physiological action potential-induced, Ca^2+^-triggered release pathway. Their data characterize two independently Ca^2+^-triggered pathways of synaptic vesicle fusion at central synapses that likely perform distinct physiological functions.

Of the three specific receptors for α-LTX, neurexin binds to α-LTX only in the presence of extracellular Ca^2^^+^, while latrophilin and protein tyrosine phosphatase σ bind to α-LTX even in the absence of extracellular Ca^2^^+^. Because the contribution of protein tyrosine phosphatase σ is small [5], latrophilin is a key receptor of Ca^2^^+^-independent secretion by α-LTX. The main exogenous ligand of latrophilin 1 is α-LTX [61]. The precise mechanism of latrophilin-mediated exocytosis in response to α-LTX is not well understood [62]. Hiramatsu *et al.* [63] transfected mast cells, typical non-neuronal secretory cells, with latrphilin and investigated the effects of α-LTX on exocytotic release from the transfected mast cells. It was found that α-LTX caused intracellular Ca^2^^+^ to increase and lead to exocytosis in the presence of extracellular Ca^2^^+^. However, neither Ca^2^^+^ increase nor exocytosis was observed in the absence of extracellular Ca^2^^+^. These observations indicate that, in the presence of extracellular Ca^2^^+^, latrophilin-bound α-LTX can work as a Ca^2^^+^ ionophore. In addition, α-LTX was found to affect the signal transduction in mast cells by phosphorylating SNARE proteins including SNAP-23, syntaxin-4, and VAMP-8 through PKC (protein kinase C)-dependent and PKC-independent pathways. These results demonstrate that, in the presence of extracellular Ca^2+^, the latrophilin-mediated toxic effect of α-LTX can be exerted through the elevation of intracellular Ca^2^^+^ and the phosphorylation of SNARE proteins, which expands our understanding of the latrophilin-mediated action mechanism of α-LTX.

In the last decades, latrotoxins, particularly α-LTX, from the venom of the black widow spider have been extensively employed as agent tools to investigate the molecular mechanism involved in the regulation of neurotransmitter release. The resulting achievements have greatly improved our understanding of the synaptic transmission [5]. Besides, Mesngon and McNutt [64] have probed the possibility of using α-LTX as a therapeutic agent. They developed embryonic stem cell-derived neurons (ESNs) as a therapeutic research platform and evaluated the potential for α-LTX to antagonize botulinum poisoning. The botulinum neurotoxins (BoNTs) are the most poisonous substances known and exhibit zinc-dependent proteolytic activity against members of the core synaptic membrane fusion complex, preventing neurotransmitter release and resulting in neuromuscular paralysis. It was demonstrated that α-LTX attenuated the severity or duration of BoNT-induced paralysis in neurons. Treatment of BoNT-intoxicated ESNs with α-LTX rescued full-length SNAP-25 expression. This is the first demonstration of a successful therapeutic application of α-LTX and suggests that α-LTX treatment may provide the basis for a new class of therapeutic approach to BoNT intoxication.

## 5. Toxins outside Venom Glands

Different from many other venomous animals including snakes and some other spider species that have toxins only in their venom glands, widow spiders not only have venom toxins, but also contain non-venom toxins [18,19,20,21]. Although there were sporadic reports on the relevant studies from very early times, it was not until recently that the toxins outside the venom glands underwent a relatively systematic investigation.

### 5.1. Egg Toxicity

By using multiple physiological and biochemical strategies, Yan *et al.* [65] characterized the aqueous extract of the eggs of *L. tredecimguttatus.* The eggs were demonstrated to be rich in high-molecular-mass proteins and the peptides were below 5 kDa, showing multiple hydrolase activities and neurotoxicities towards mammals and insects. For investigating the possible relationship of proteins in the eggs with the egg toxicity, Li *et al.* [66] analyzed the protein composition of the eggs using proteomic strategies and compared it with that of the spider’s venom. The proteins of eggs were shown to be primarily distributed in the molecular mass range of higher than 55 kDa as well as around 34 kDa, having high abundance proteins with molecular masses of about 60 kDa and 130 kDa. A total of 157 proteins were identified from the egg extract which are involved in important cellular functions and processes including catalysis, transport, and metabolism regulation. Comparison indicated that the protein composition of eggs is more complex than that of venom, and there are only a few similarities between the protein compositions of the two materials. No known typical black widow spider venom proteins were found in the egg extract, suggesting that the eggs have their own distinct toxic mechanism (Table 3).

**Table 3:** Comparison of the numbers of proteins identified from the eggs and venom of *L. tredecimguttatus* ^a^.

Classification	Egg Extract (%)	Venom (%) ^b^
(i) known typical black widow spider venom proteins	0	10 (8.2)
(ii) hydrolases	12 (7.6)	13 (10.7)
(iii) other enzymes	51 (32.5)	20 (16.4)
(iv) proteins of unknown function	14 (8.9)	25 (20.5)
(v) proteins with binding function	44 (28.0)	23 (18.9)
(vi) other proteins	36 (22.9)	31 (25.4)
Total	157 (100)	122 (100)

^a^, Li *et al.* [66]; ^b^, Duan *et al.* [27].

### 5.2. Toxins Purified from the Eggs

For obtaining more detailed information on the egg toxicity of the black widow spider, much earlier efforts had been made to purify and characterize the toxic components from the spider eggs; however, the work was unsuccessful or incomplete [20,21]. Recently, four proteinaceous bioactive components were purified and preliminarily characterized from the eggs of the black widow spider (*L. tredecimguttatus*); they were named Latroeggtoxin-I to Latroeggtoxin-IV [67,68,69]. Latroeggtoxin-I has a molecular mass of 23.8 kDa and can block the neuromuscular transmission in isolated mouse phrenic nerve-hemidiaphragm preparations completely in a reversible manner. BLAST analysis using the determined *N*-terminal sequence of the protein demonstrated that no significant similarity to the existing proteins was found, indicating that Latroeggtoxin-I is a novel neurotoxic protein purified from the eggs of the black widow spider [67]. After being abdominally injected into mice and *P. americana*, Latroeggtoxin-II, with a molecular mass of 28.7 kDa, made the animals, especially *P. Americana*, display a series of symptoms of poisoning, including becoming depressed, moving slowly, and lagging in response. Preliminary electrophysiological experiments demonstrated that Latroeggtoxin-II selectively inhibits tetrodotoxin-resistant Na^+^ channel currents in rat dorsal root ganglion neurons, without significant effect on the tetrodotoxin-sensitive Na^+^ channel currents. Using multiple proteomic strategies, Latroeggtoxin-II was shown to have only a few similarities to the existing proteins in the databases, suggesting that it is also a novel protein isolated from the eggs of the black widow spider [68]. Preliminary analyses indicate that Latroeggtoxin-III has a molecular mass of about 36.0 kDa and exhibits neurotoxicity against cockroaches but has no obvious effect on mice, suggesting that Latroeggtoxin-III, different from Latroeggtoxin-I and -II, is an insect-specific toxin. When utilizing the determined *N*-terminal sequence of Latroeggtoxin-III to perform a homology analysis using the protein BLAST program, Latroeggtoxin-III was demonstrated to be a proteolytically cleaved product of vitellogenin [69]. Different from Latroeggtoxin-I to -III, Latroeggtoxin-IV is a broad-spectrum antibacterial peptide of 3.6 kDa, showing inhibitory activity against all the five tested species of bacteria (*S. aureus*, *S.* *typhimurium*, *B. subtilis*, *E. coli*, *P.* *aeruginosa*), with the highest activity against *S. aureus* [69].

### 5.3. Spiderling Toxicity

The early preliminary studies found that the spiderlings of the black widow spider (*L. tredecimguttatus*) had obvious toxicity to animals [20,21]. To further probe the black widow spiderling toxicity, Peng *et al.* [70] performed a systematical analysis of the aqueous extract of newborn black widow spiderlings. The extract was shown to contain 69.42% of proteins varying in molecular masses and isoelectric points. Abdominal injection of the extract into mice and cockroaches caused obvious poisoning symptoms as well as death, with LD_50_ being 5.30 mg/kg in mice and 16.74 μg/g in *P. americana*. Electrophysiological experiments indicated that the extract at a concentration of 10 μg/mL could completely block the neuromuscular transmission in isolated mouse phrenic nerve-hemidiaphragm preparations within 21.0 ± 1.5 min, and 100 μg/mL extract could inhibit a certain percentage of voltage-activated Na^+^, K^+^, and Ca^2^^+^channel currents in rat dorsal root ganglion neurons. These results demonstrate that the spiderlings are rich in neurotoxic and other bioactive components, which play important roles in the spiderling toxicity.

### 5.4. Implications of the Toxins outside Venom Glands

Why the spiderlings and even the eggs of the black widow spider, like the venom glands, have evolved toxic components is an interesting question. It is speculated that the existence of such components provides a certain protection from some greedy arthropods, which is supported by the report of Russell *et al.* [71]. They demonstrated that *Latrodectus* egg toxins have deleterious effects on the web-building activity of *A. diadematus*. The web-building activity of the spiders receiving 3–5 g/kg body weight was abnormal and one spider receiving 1 g/kg body weight died 6 h after feeding. On the other hand, antimicrobial components may play a dual role in spider-prey interaction, functioning both in the prey capture strategy as well as in protecting the spider from potentially infectious organisms arising from prey ingestion. The adult female black widow spiders usually suspend their egg sacs from the ceiling deep in the retreat, where it is often dark and humid and suitable for the breeding of pathogenic microorganisms. There must be specific mechanisms to protect the eggs and spiderlings. Thus, it is speculated that the antibacterial components may play important roles in protecting the eggs and newborn spiderlings from some pathogenic microorganisms.

## 6. Conclusions and Outlook

The widow spider is a peculiar animal not only for its being one of the most poisonous animals, but also for its toxin distribution throughout its body, even in the eggs and newborn spiderlings. Up until now, research on latrotoxins, particularly the α-LTX, has been extensive; however, the mechanism of action of the toxins has not been fully understood and is still the research hotspot in the relevant field. On the other hand, research on the toxins outside the venom glands is relatively limited. Although there has been a series of studies investigating the toxicity outside the venom glands of the spider and several proteinaceous toxic components were purified and preliminarily characterized, the shortage of natural resources of the toxins limits the detailed structure-function analysis of the toxins. Therefore, gene cloning and heterologous expression might be necessary in further research.
